# CMTM3 promotes adipocyte differentiation by regulating PPARγ in 3T3-L1 cells

**DOI:** 10.1016/j.gendis.2025.101699

**Published:** 2025-05-30

**Authors:** Yanni Zhao, Meiyu Piao, Yuankuan Li, Sung Ho Lee, Seung-Joo Nho, Yujian Jin, Chang-Yeol Yeo, Kwang Youl Lee

**Affiliations:** a School of Pharmaceutical Sciences, Wenzhou Medical University, Wenzhou, Zhejiang 325035, China; b College of Pharmacy & Research Institute of Pharmaceutical Sciences, Chonnam National University, Gwangju 61186, Republic of Korea; c Department of Life Science, Ewha Woman's University, Seoul 03760, Republic of Korea

CKLF-like MARVEL transmembrane domain containing 3 (CMTM3) has been reported to suppress tumors significantly in various cancer types. However, the molecular biological functions of CMTM3 outside of cancer remain largely unknown. In our study, we aim to discover novel functions of CMTM3 in adipocytes and elucidate the molecular mechanism through which CMTM3 functions in adipogenesis. We observed a significant positive role of CMTM3 during adipocyte differentiation in both gain- and loss-of-function experiments. Mechanically, co-immunoprecipitation and luciferase assay were applied to confirm the relationship between peroxisome proliferator-activated receptor gamma (PPARγ) and CMTM3. Furthermore, the functional study of CMTM3 mutations facilitated our understanding of the regulation of adipogenesis. Our findings reveal a new biological function of CMTM3 in adipogenesis and shed light on its potential as a molecular target in obesity therapy.

Recent research hinted at the relationship between CMTM3 and PPARγ, whereby the pro-tumorigenic effects of CMTM3 in hepatocellular carcinomas involve the modulation of PPARγ.^1^ We validated the role of CMTM3 in 3T3-L1 adipocyte differentiation using a gain-of-function experiment. *CMTM3* overexpression increased oil red O staining intensity and lipid accumulation of 3T3-L1 adipocyte differentiation (Fig. 1A; Fig. S1B). Meanwhile, *CMTM3* overexpression improved the mRNA levels of *Pparg* and CCAAT-enhancer binding protein alpha (*Cebpa*) after 8 days of differentiation, but not *Cebpb* (Fig. 1A). Even though low CMTM3 expression was maintained during adipogenesis (Fig. S1A), further suppressed CMTM3 expression significantly inhibited adipogenesis (*CMTM3* knockdown using small hairpin RNA in 3T3-L1 cells) (Fig. 1B; Fig. S2A, B). The mRNA levels of *Pparg* and *Cebpa* were also attenuated by CMTM3 silencing at the endpoint of adipogenesis, but not *Cebpb* (Fig. 1B; Fig. S2A). To further confirm which stage of adipogenesis was regulated by CMTM3, we examined the levels of the adipogenesis-related markers at the indicated times (day 0, 2, 4, 6, and 8). We found that the master regulator of PPARγ was affected by both CMTM3 and shCMTM3 transfection in the early and late stages of adipogenesis (Fig. S1C, D, 2C). These results suggest that CMTM3 plays an important role in adipogenesis and mainly targets PPARγ.

**Figure 1:**
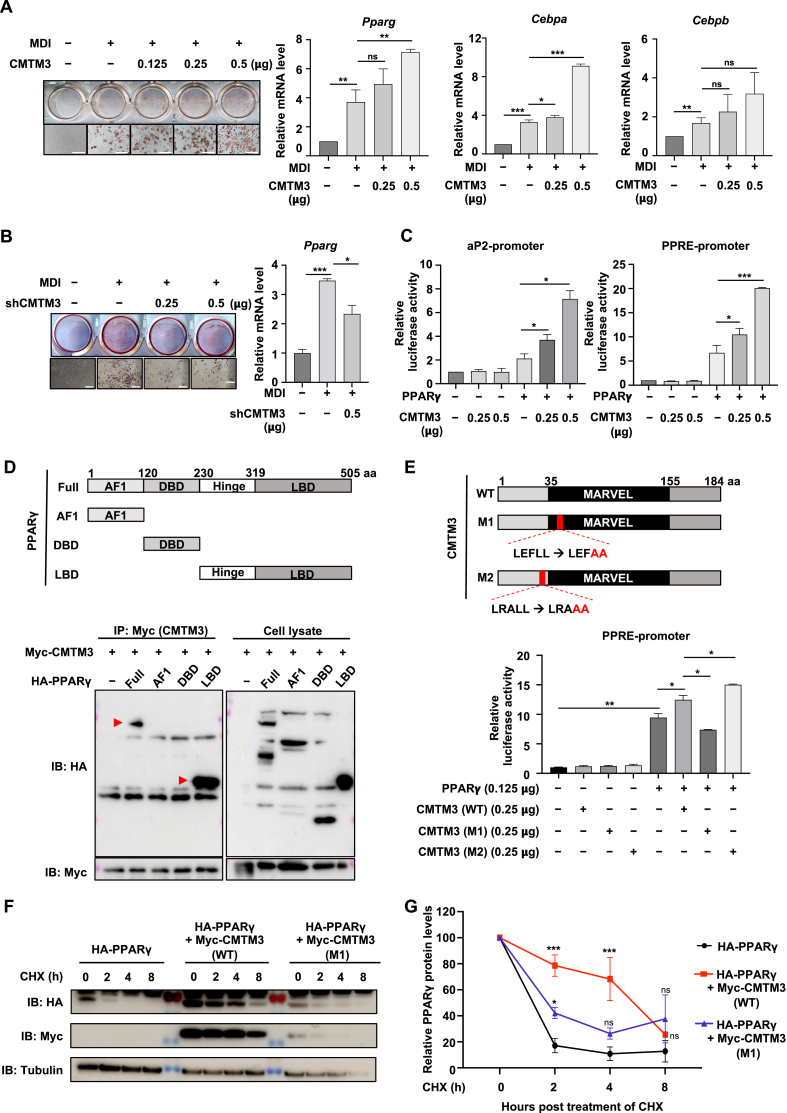
CMTM3 is a positive regulator during adipocyte differentiation. **(A)** Pre-adipocyte 3T3-L1 cells were transiently transfected with increasing amounts of CMTM3 (0.125, 0.25, 0.5 μg) and differentiated with MDI (0.5 mM of 3-isobutyl-1-methylxantine, 1 μM of dexamethasone, and 10 μg/mL of insulin) until day 8. An empty vector was used as the transfection control. Oil red O staining was used to visualize differentiated adipocytes. Scale bar = 100 μm. Quantitative reverse transcription PCR was used to determine the mRNA levels of *Pparg*, *Cebpa*, and *Cebpb*. **(B)** Pre-adipocyte 3T3-L1 cells were transiently transfected with shCMTM3 (0.25, 0.5 μg) and differentiated with MDI until day 8. A pSuper empty vector was used as the transfection control. Oil red O staining was used to visualize differentiated adipocytes. Scale bar = 100 μm. Quantitative reverse transcription PCR was used to determine the mRNA levels of *Pparg*. **(C)** HEK 293 cells were transfected with PPARγ (0.125 μg) and with or without CMTM3 (0.25, 0.5 μg). The aP2- and PPRE-promoter activity was assessed using the luciferase assay. **(D)** The schematic diagram of PPARγ deletion. HEK 293 cells were transfected with an indicated combination of CMTM3 and the deleted-PPARγ form. Co-immunoprecipitation was performed using anti-Myc, and immunoblotting (IB) was conducted using anti-Myc and anti-HA. F, PPARγ full-length; AF1, activation function 1; DBD, DNA binding domain; LBD, ligand-binding domain. **(E)** The schematic diagram of the CMTM3 point mutation. M1, mutation of LEFLL, located in the MARVEL domain; M2, mutation of LRALL, located in the N-terminal domain. HEK 293 cells were transfected with an indicated combination of PPARγ (0.125 μg) and CMTM3 (WT, M1, and M2) (0.25 μg**)**. The PPRE promoter activity was performed using the luciferase assay. **(F, G)** HEK 293 cells were transfected with an indicated combination of HA-PPARγ and Myc-CMTM3 (WT, M1) for 24 h, and then treated with cycloheximide (40 μg/mL) for the indicated time (0, 2, 4, 8 h). The protein intensities (PPARγ) were quantified using Image J Version 1.52 k. Data were expressed as mean ± standard error of the mean of at least three experiments. ns, not significant. ∗*p* < 0.05, ∗∗*p* < 0.01, and ∗∗∗*p* < 0.001.

Previous research showed that PPARγ is an adipocyte-specific nuclear hormone receptor crucial for adipocyte development *in vitro* and *in vivo*.^2^ First, we examine the association between CMTM3 and PPARγ transcriptional activity. *PPARγ* overexpression alone increased those promoter activities, while they were further increased by co-transfection with CMTM3 (Fig. 1C). Moreover, overexpression of *CMTM3* effectively up-regulated rosiglitazone-induced PPARγ transcriptional activity (Fig. S3A). Conversely, *CMTM3* knockdown suppressed the transcriptional activity of PPARγ in the absence and presence of rosiglitazone (Fig. S3B). Secondly, we confirmed the interaction between CMTM3 and PPARγ. The co-immunoprecipitation assay showed that CMTM3 and PPARγ could interact with each other (Fig. S3C). Furthermore, we generated a series of PPARγ deletion mutants to examine which domain was responsible for interacting with CMTM3. The co-immunoprecipitation assay showed that the PPARγ ligand binding domain (LBD) interacted with CMTM3 (Fig. 1D). Taken together, these results show that CMTM3 binds to and increases PPARγ transcriptional activity.

To gain insight into the mechanism of CMTM3 in regulating PPARγ′s transcriptional activity, we constructed a series of CMTM3 mutants. The co-immunoprecipitation assay showed that all the domain mutants for CMTM3 interacted with PPARγ, and they all contained the MARVEL domain (Fig. S4A). Furthermore, the luciferase assay showed that no significant difference was observed in the transcriptional activity of PPARγ between the domain mutant and wild type (WT) of CMTM3 (Fig. S4B). To further elucidate the importance of the MARVEL domain, we cloned the MARVEL cutting form as shown in Figure S4C. As the data showed, the mutation in the MARVEL domain cannot interact with PPARγ (Fig. S4C). These results indicate that the MARVEL domain in CMTM3 has an important role in the interaction between CMTM3 and PPARγ. Interestingly, recent research has shown that CMTM3 has two “LXXLL” motifs (where L represents leucine and X represents any amino acid) at the N-terminal and MARVEL domains, and these special structures are often present in steroid receptor binding proteins, such as the androgen receptor and nuclear receptor.^3^ Thus, we designed CMTM3 point mutations at the LXXLL motif, and determined whether the LXXLL motif in CMTM3 was involved in regulating the transcriptional activity of PPARγ (Fig. 1E). The interaction between CMTM3 and PPARγ was not affected by the introduction of a point mutation (Fig. S4D). However, the transcriptional activity of PPARγ was significantly suppressed by the point mutation (M1) in CMTM3, located in the MARVEL domain. The other point mutation (M2) in CMTM3 did not affect the transcriptional activity (Fig. 1E). Overall, these results demonstrate that the MARVEL domain in CMTM3, particularly its LXXLL motif, plays a crucial role in regulating PPARγ activity.

Even though we fully indicated the relationship between CMTM3 and PPARγ as described above, the mechanism by which CMTM3 regulates PPARγ remains unclear. To further elucidate the regulatory mechanism, we investigated whether the protein stability of PPARγ was affected by CMTM3. Firstly, the exogenous protein levels of PPARγ were increased by *CMTM3* overexpression in a dose-dependent manner (Fig. S4E). Moreover, the protein levels of PPARγ were inhibited by the CMTM3-M1 mutation. However, the M2 mutation showed a mimicry effect compared with the WT (Fig. S4F). To provide more evidence that CMTM3 modulates PPARγ protein levels, we analyzed the half-life of PPARγ with the CMTM3-WT or M1 mutation form. As the previous research indicated, PPARγ is a short-lived protein and is rapidly degraded after cycloheximide is added.^4^ Interestingly, overexpression of the *CMTM3-WT* increased the PPARγ half-life from 2 h to beyond 4 h, while the effects of the M1 mutation were lower than the WT (Fig. 1F, G). These results suggest that CMTM3 improves the protein stability of PPARγ.

In conclusion, the present study found a novel biological function of CMTM3 in adipogenesis. It provides evidence that CMTM3 has a significant role in adipogenesis and obesity, in addition to a tumor suppressor function in cancer. Additionally, we show that CMTM3 functions as an upstream factor of PPARγ, which regulates both protein stability and transcriptional activity. Mechanically, CMTM3 works as a positive regulator through its LXXLL motif, which is located in the MARVEL domain of CMTM3. This study provides a massive hint for investigating the function of CMTM3.

## CRediT authorship contribution statement

**Yanni Zhao:** Formal analysis, Investigation, Methodology, Visualization, Writing – original draft. **Meiyu Piao:** Formal analysis, Investigation, Methodology, Visualization, Writing – original draft, Writing – review & editing. **Yuankuan Li:** Formal analysis, Investigation, Methodology, Writing – review & editing. **Sung Ho Lee:** Formal analysis, Investigation, Methodology, Writing – review & editing. **Seung-Joo Nho:** Formal analysis, Investigation, Methodology, Writing – review & editing. **Yujian Jin:** Formal analysis, Investigation, Methodology, Writing – review & editing. **Chang-Yeol Yeo:** Investigation, Resources, Supervision, Validation, Writing – review & editing. **Kwang Youl Lee:** Funding acquisition, Investigation, Project administration, Resources, Supervision, Validation, Writing – review & editing.

## Data availability

The datasets analyzed during the current study are available from the corresponding author upon reasonable request.

## Funding

This work was supported by a National Research Foundation of Korea (NRF) grant funded by the Korean government (MSIT) (No. 2019R1A5A2027521).

## Conflict of interests

The authors declared no conflict of interests.
