# Supplementary material for: CMTM3 promotes adipocyte differentiation by regulating PPARγ in 3T3-L1 cells
Source: Genes Dis. 2025 May 30;12(6):101699. doi: 10.1016/j.gendis.2025.101699 (PMC12280988; doi:10.1016/j.gendis.2025.101699)
Supplement: Multimedia component 1 [file mmc1.docx]

**Materials and Methods**

**Cell culture and adipocyte differentiation**

Mouse preadipocyte 3T3-L1 cells and human embryonic kidney HEK 293 cells were maintained in Dulbecco’s Modified Eagle Medium (DMEM) containing 10% fetal bovine serum (FBS) and 1% antibiotic–antimycotics at 37 °C with 5% CO_2_ incubator. For 3T3-L1 cell differentiation, fully confluent cells were replaced in a differentiation medium, which contained DMEM, 10% FBS, and differentiation cocktail (MDI) consisting of 0.5 mM of 3-isobutyl-1-methylxantine, 1 μM of dexamethasone, and 10 μg/mL of insulin for 2 days. After two days, the differentiation medium was replaced with 10% FBS/DMEM containing insulin (10 μg/mL of insulin) every two days until terminal differentiation (day 8). On day 8, 3T3-L1 preadipocytes were fully differentiated into mature adipocytes, which were used for further experiments. All the reagents used for cell culture and differentiation are presented in Table S1.

**Plasmids and transfection**

HA-tagged PPARγ (full length, 1–120 aa, 121–230 aa, 231–505 aa), Myc-tagged CMTM3 wild type (WT), Myc-tagged CMTM3 deletion mutants (MT1, MT2, MT3), and Myc-tagged CMTM3 point mutants (M1, M2) were constructed in a CMV promoter-derived mammalian expression vector (pCS4-3HA, and pCS4-3Myc), respectively. All overexpression plasmids including WT, mutant, and deletion, were constructed using the primer sets in Table S2. The polyethyleneimine (Polysciences Inc, Warrington, PA, USA)-mediated transient transfection method was used for HEK 293 and 3T3-L1 cell transfection, and the transfection controls were established using empty vectors.

**Small hairpin RNA (shRNA) construction**

To construct the CMTM3 knockdown (shCMTM3), a 21-base pair (bp) sequence (GCC GTT TAC TTC CTC TTT GCT) of the mouse *CMTM3* gene was used to synthesize the small hairpin RNA (shRNA). The shRNA of CMTM3 was constructed in pSuper retro puro vectors (Oligoengine, Seattle, WA, USA), and the transfection controls were established using pSuper retro puro vectors.

**Oil Red O staining**

Oil Red O staining was used to visualize the lipid droplet accumulation in mature 3T3-L1 adipocytes on day 8. Briefly, the fully differentiated cells were prepared with PBS wash and 10% formalin fixation. Subsequently, the fixed cells were stained with Oil Red O working solution (0.5% Oil Red O stock solution in ddH_2_O) at room temperature for 1 hour. The stained cells were observed under an inverted light microscope. For lipid accumulation analysis, isopropanol was used to dissolve the stained cells, and the absorbance was measured using an Epoch microplate reader at 510 nm.

**Immunoblotting (IB) and co-immunoprecipitation (co-IP)**

Cold-lysis buffer containing protease inhibitor cocktails (25 mM HEPES pH 7.4, 150 mM NaCl, 1% NP-40, 0.25% sodium deoxycholate, 10% glycerol, 25 mM sodium fluoride, 1 mM EDTA, 1 mM Na_3_VO_4_, 250 µM PMSF, 10 µg/mL leupeptin, 10 µg/mL pepstatin, and 10 µg/mL aprotinin) was used for total protein extraction from HEK 293 and 3T3-L1 cells. Each 20 µg of protein was separated by sodium dodecyl sulfate-polyacrylamide gel electrophoresis (SDS-PAGE) and electro-transferred to polyvinylidene difluoride membranes (PVDF; Immobilon-P; Millipore, Burlington, MA, USA). The PVDF membranes were blocked in 5% skim milk and subsequently incubated with appropriate antibodies at 4 ℃ overnight. All antibodies used for IB as presented in Table S3. After washing and incubating with secondary antibodies, which are HRP-conjugated mouse and rabbit at RT for 1 h. Immobilon Western Chemiluminescent HRP Substrate (WBKLS0500, Millipore) was used to visualize protein on an Amersham^TM^ ImageQuant^TM^ 800 biomolecular imager. For co-IP, HEK 293 cells were transfected with an indicated combination of PPARγ (WT, deletion), CMTM3 (WT, MT1, MT2, MT3), and CMTM3 (M1, M2) for 48 h. After transfection, whole cell lysates were extracted by cold-lysis buffer, and 200 µg of lysates were prepared and incubated overnight with the anti-Myc or anti-HA antibody. The complexed samples were further incubated with protein A-Sepharose CL-4B (#17096303; GE Healthcare Life Sciences) at 4 ˚C for 1 h, followed by IB.

**Protein stability assay**

The indicated combination of HA-PPARγ (0.125 µg) and Myc-CMTM3 (WT, M1) (0.25 µg) are transfected into the HEK 293 cells for 48 h. The transfected cells were treated with cycloheximide (CHX; 40 µg/mL; C1988, Sigma-Aldrich), and harvested lysates at indicated times after treatment of CHX (0 h, 2 h, 4 h, and 8 h). Then, the harvested lysates were used to perform IB.

**Reverse Transcription Followed by Quantitative Polymerase Chain Reaction (RT-qPCR)**

TRIzol reagent (TaKaRa, Tokyo, Japan) was used for total RNA isolation from 3T3-L1 cells. Each RNA (1 µg) was reverse-transcribed to cDNA using Oligo dT primers and GoScript^TM^ Reverse Transcription System (Promega, Madison, WI, USA), according to the manufacturer’s instructions. RT-qPCR was performed using the QuantStudio^TM^3 RT-PCR (Applied Biosystems, USA), and the primer sequences used for PCR are as follows in Table S4. The relative amount of each target transcript was normalized to *Gapdh*.

**Luciferase reporter assay**

HEK 293 and 3T3-L1 cells were co-transfected with β-galactosidase (pCMV-β-gal) (0.05 µg), luciferase reporter plasmid (aP2-promoter, PPRE-promoter) (0.2 µg), PPARγ (0.125 µg) and increasing amounts of CMTM3 expression plasmids (0.25 µg, 0.5 µg) or 0.25 µg of shCMTM3, deletion, and point mutation form as indicated combination. After transfection, the promoter activities were analyzed using the Luciferase Reporter Assay Kit (Promega) according to the manufacturer’s instructions. Transfection efficiency was normalized using β-gal activity. Experiments were performed in triplicate and repeated at least three times.

**Statistical analysis**

Statistical analysis was performed using GraphPad Prism v8.0 Software (San Diego, CA, USA). One-way ANOVA was followed by a multiple comparisons test. All experiments were repeated at least three times. The results are expressed as the mean ± SEM, and a *p*-value < 0.05 was considered significant.

**Supplementary tables**

**Table S1.** The reagent information

| Reagent | Catalog | Company |
| --- | --- | --- |
| DMEM | 12100-046 | Gibco^TM^, Carlsbad, CA, USA |
| Fetal bovine serum (FBS) | S001-07 | Welgene Inc, Daegu, Korea |
| antibiotic–antimycotics | 15240062 | Gibco^TM^, Carlsbad, CA, USA |
| 3-isobutyl-1-methylxantine (IBMX) | I5879 | Sigma-Aldrich, St. Louis, MO, USA |
| Insulin | I9278 | Sigma-Aldrich, St. Louis, MO, USA |
| Dexamethasone | D4902 | Sigma-Aldrich, St. Louis, MO, USA |

**Table S2.** The primers used for constructing the plasmids.

| Gene name | | Sequence |
| --- | --- | --- |
| PPARγ_full length | Forward | CCG CTC GAG ATG GGT GAA ACT CTG GGA |
|  | Reverse | CTA GCT AGC CTA ATA CAA GTC CTT GTA |
| PPARγ_1–120 aa | Forward | CGG AAT TCA ATG GGT GAA ACT CTG GGA |
|  | Reverse | CCG CTC GAG CTT TTC AGA ATA ATA AGG |
| PPARγ_121–230 aa | Forward | CGG AAT TCA ACC CAG CTC TAC AAC AGG |
|  | Reverse | CCG CTC GAG GAT ATC ACT GGA GAT CTC |
| PPARγ_231–505 aa | Forward | CGG GAT CCC GGA CCA GCT GAA CCC AGA G |
| CMTM3-WT | Forward | GCC GAA TTC ATG GCC CCC GGA CGC AGA |
|  | Reverse | CGG CTC GAG TTA GTC AGA GTC TGA GTC |
| CMTM3-MT1 | Reverse | CGG CTC GAG GGC CAC TTC ATT AAA GA |
| CMTM3-MT2 | Forward | GCC GAA TTC ATT CCT CTG CTC TCT CA |
| CMTM3 1–92 aa | Reverse | CCG CTC GAG TTT GTC ATT CAG CTG CAT |
| CMTM3 93–184 aa | Forward | CGG AAT TCA TGG CAG GGT TTG TGC TGG |
| CMTM3-M1 | Forward | ACT CCG AGC CGC AGC ACC CGC GCG CGC CTT |
|  | Reverse | AAG GCG CGC GCG GGT GCT GCG GCT CGG AGT |
| CMTM3-M2 | Forward | CTG GAG TTT GCG GCG GCC GTT TAC TTC CTC |
|  | Reverse | GAG GAA GTA AAC GGC CGC CGC AAA CTC CAG |

**Table S3.** The antibody information.

| Antibody name | Catalog | Company |
| --- | --- | --- |
| CMTM3 | H00123920-B01P | Novus Biologicals, Littleton, CO, USA |
| C/EBPβ | SC-150 | Santa Cruz Biotechnology, Santa Cruz, CA, USA |
| C/EBPα | SC-61 | Santa Cruz Biotechnology, Santa Cruz, CA, USA |
| PPARγ | 2435 | Cell signaling Technology, Danvers, MA, USA |
| Myc | 9E10 | Roche Applied Science, Basel, Switzerland |
| HA | 12CA5 | Roche Applied Science, Basel, Switzerland |
| α-tubulin | sc-23948 | Santa Cruz Biotechnology, Santa Cruz, CA, USA |

**Table S4.** The RT-qPCR primer information.

| Gene name | | Sequence |
| --- | --- | --- |
| *Pparg* | Forward | 5’-ATC TTT GGT CTG GCT CCC ATG-3’ |
|  | Reverse | 5’-TTT CCC GTT CAC CGT CCA C-3’ |
| *Cebpb* | Forward | 5’-AGC CCC TAC CTG GAG CCG CT-3’ |
|  | Reverse | 5’-GCG CAG GGC GAA CGG GAA AC-3’ |
| *Cebpa* | Forward | 5’-TGC TGG AGT TGA CCA GTG AC-3’ |
|  | Reverse | 5’-AAA CCA TCC TCT GGG TCT CC-3’ |
| *Cmtm3* | Forward | 5’-GCC GAG TCG GGT CTT TCA TTC-3’ |
|  | Reverse | 5’-GAG GAA GTA AAC GGC CAA CAG-3’ |
| GAPDH | Forward | 5’-AGG TCG GTG TGA ACG GAT TTG-3’ |
|  | Reverse | 5’-GGG GTC GTT GAT GGC AAC A-3’ |
