# Supplementary material for: CMTM3 promotes adipocyte differentiation by regulating PPARγ in 3T3-L1 cells
Source: Genes Dis. 2025 May 30;12(6):101699. doi: 10.1016/j.gendis.2025.101699 (PMC12280988; doi:10.1016/j.gendis.2025.101699)
Supplement: Multimedia component 2 [file mmc2.docx]

**
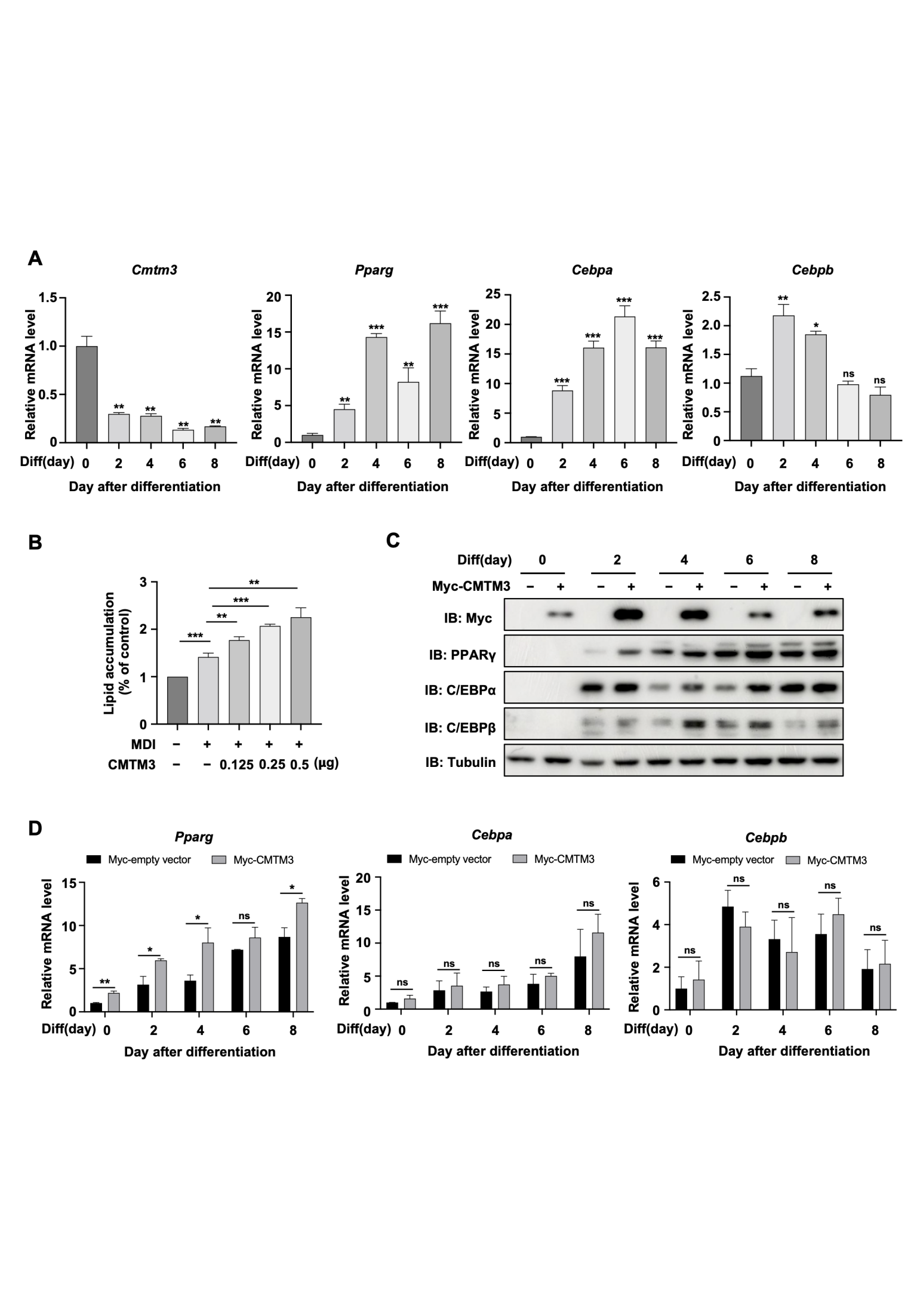
**

**Supplementary Figure 1.** CMTM3 positively regulates adipogenesis. **(A)** MDI induced the differentiation of 3T3-L1 adipocytes. RT-qPCR was used to detect the mRNA levels of *Cmtm3, Pparg, Cebpa*, and *Cebpb* at indicated days after differentiation (days 0, 2, 4, 6, and 8). GAPDH served as a loading control. **(B)** Pre-adipocyte 3T3-L1 cells were transiently transfected with increasing amounts of CMTM3 (0.125, 0.25, 0.5 µg**)**; an empty vector was used as the transfection control. The lipid accumulation was quantified. **(C, D)** The 3T3-L1 cells were transfected with or without CMTM3 (0.5 µg**)** and were induced by MDI. The samples were harvested at indicated days after differentiation (day 0, 2, 4, 6, and 8). IB was used to detect the protein levels of Myc-CMTM3, PPARγ, C/EBPα, C/EBPβ, and Tubulin. Tubulin was used as a loading control. RT-qPCR was used to determine the mRNA levels of *Pparg, Cebpa*, and *Cebpb*. GAPDH served as a loading control. Data are expressed as mean ± SEM of at least three experiments. ns, not significant. * *p* < 0.05, ** *p* < 0.01, *** *p* < 0.001.


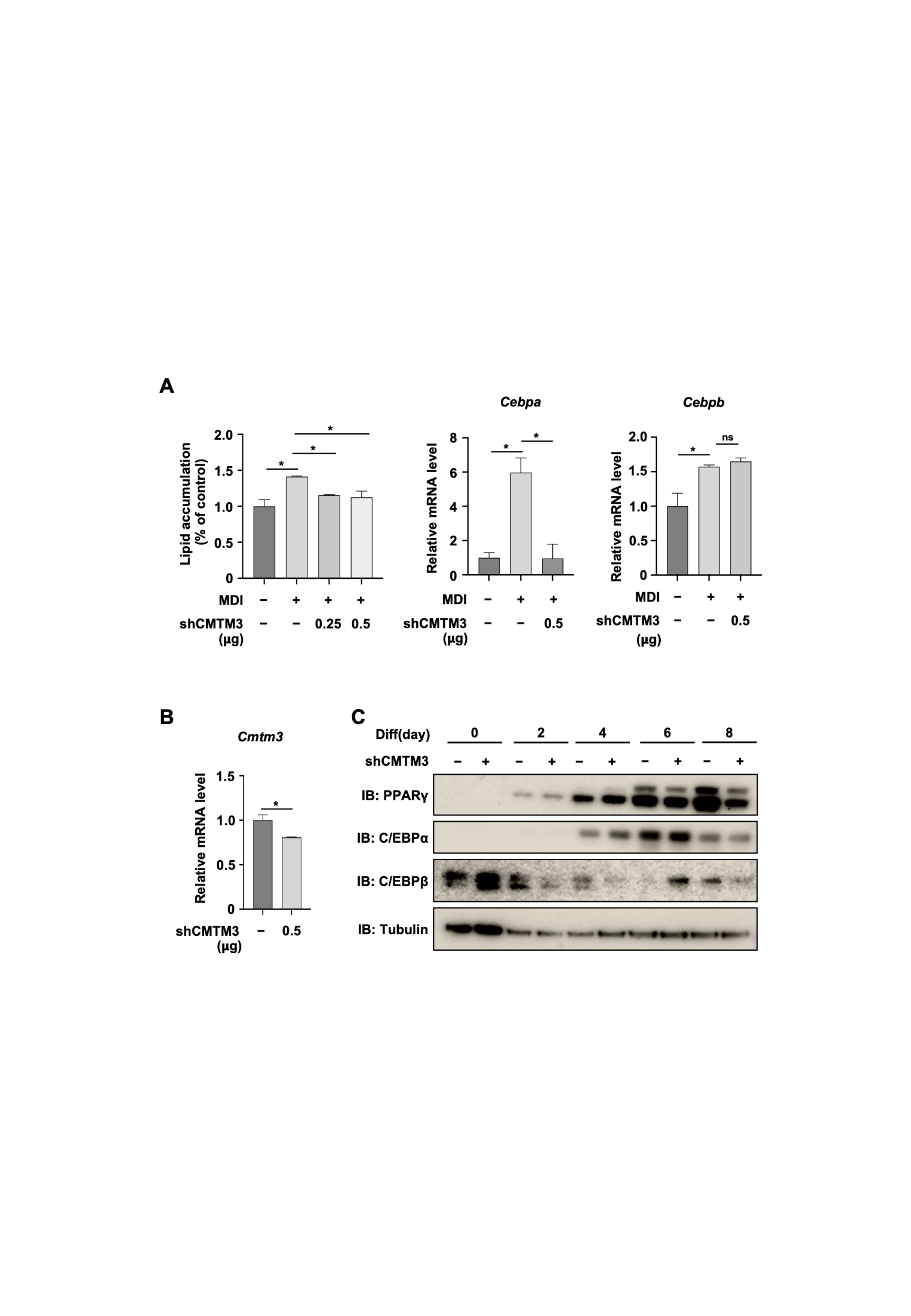


**Supplementary Figure 2.** *CMTM3* knockdown suppresses the expression of transcription factors during adipogenesis. **(A)** Pre-adipocyte 3T3-L1 cells were transiently transfected with shCMTM3 (0.25, 0.5 µg**)**; a pSuper vector was used as the transfection control. The lipid accumulation was quantified (left panel). RT-qPCR was used to determine the mRNA levels of *Cebpa*, and *Cebpb* (right panel). **(B)** The knockdown efficiency of CMTM3 was confirmed by RT-qPCR. GAPDH served as a loading control. **(C)** The 3T3-L1 cells were transfected with or without shCMTM3 (0.5 µg**)**and were induced by MDI. The protein samples were harvested at indicated days after differentiation (day 0, 2, 4, 6, and 8). IB was used to detect the protein levels of PPARγ, C/EBPα, C/EBPβ, and Tubulin. Tubulin was used as a loading control. Data are expressed as mean ± SEM of at least three experiments. ns, not significant. * *p* < 0.05.


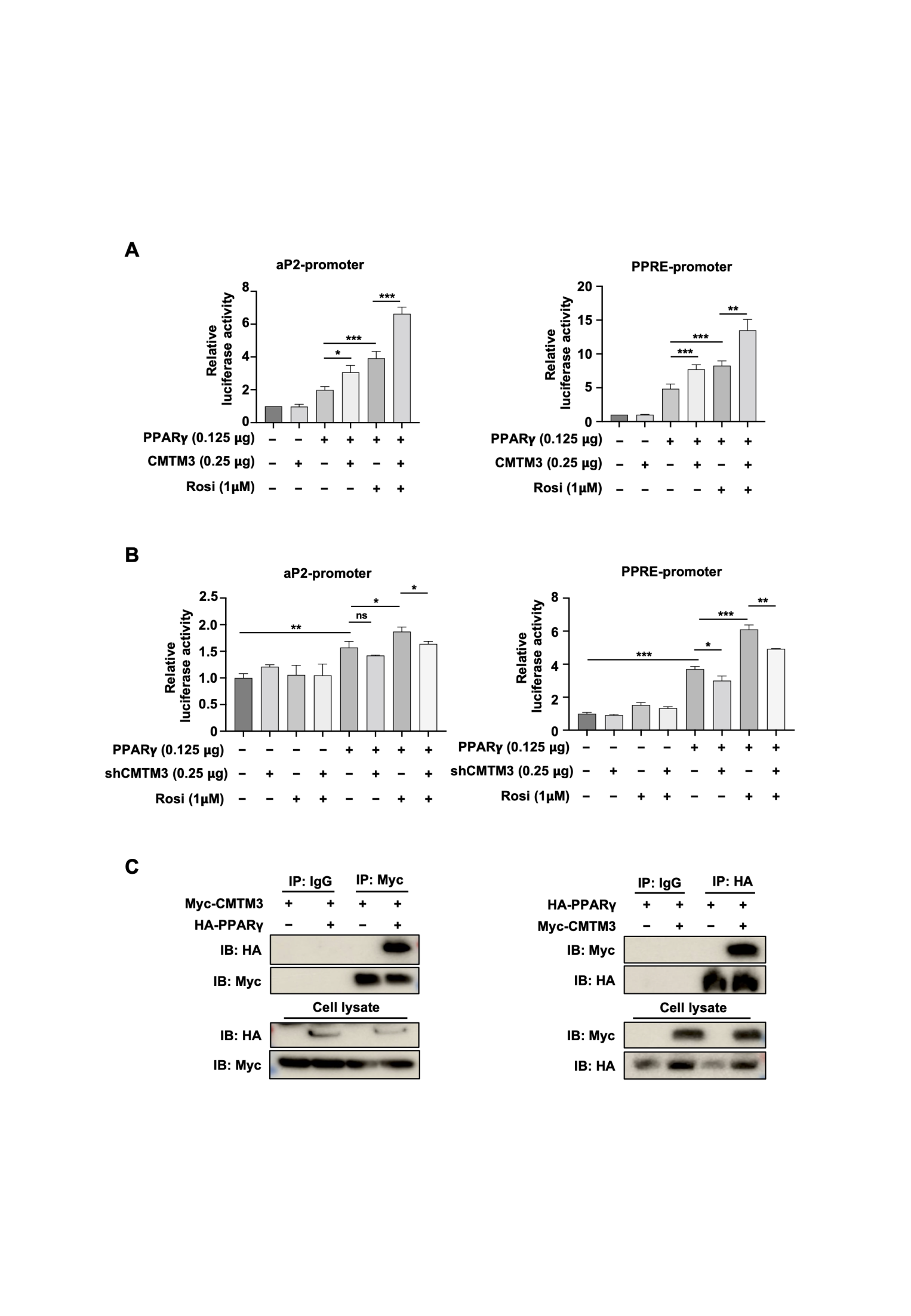


**Supplementary Figure 3.** CMTM3 directly binds to and increases PPARγ transcriptional activities. (**A)** HEK 293 cells were transfected with PPARγ (0.125 µg**)** and CMTM3 (0.25 µg**)**. After 24 h transfection, the cells were treated with or without Rosi (1 µM**)** for 16 h. The aP2- and PPRE-promoter activity was assessed by luciferase assay. (**B)** Pre-adipocyte 3T3-L1 cells were transfected with an indicated PPARγ (0.125 µg**)** and shCMTM3 (0.25 µg**)** combination and treated with or without Rosi (1 µM**)** for 16 h. The promoter activity was assessed using the luciferase assay. Data are expressed as mean ± SEM of at least three experiments. ns, not significant. * *p* < 0.05, ** *p* < 0.01, *** *p* < 0.001. **(C)** HEK 293 cells were transfected with an indicated combination of HA-PPARγ and Myc-CMTM3 expression plasmid construct. Co-IP was performed with anti-Myc and anti-HA, respectively. IB was detected using anti-Myc and anti-HA antibodies.

**
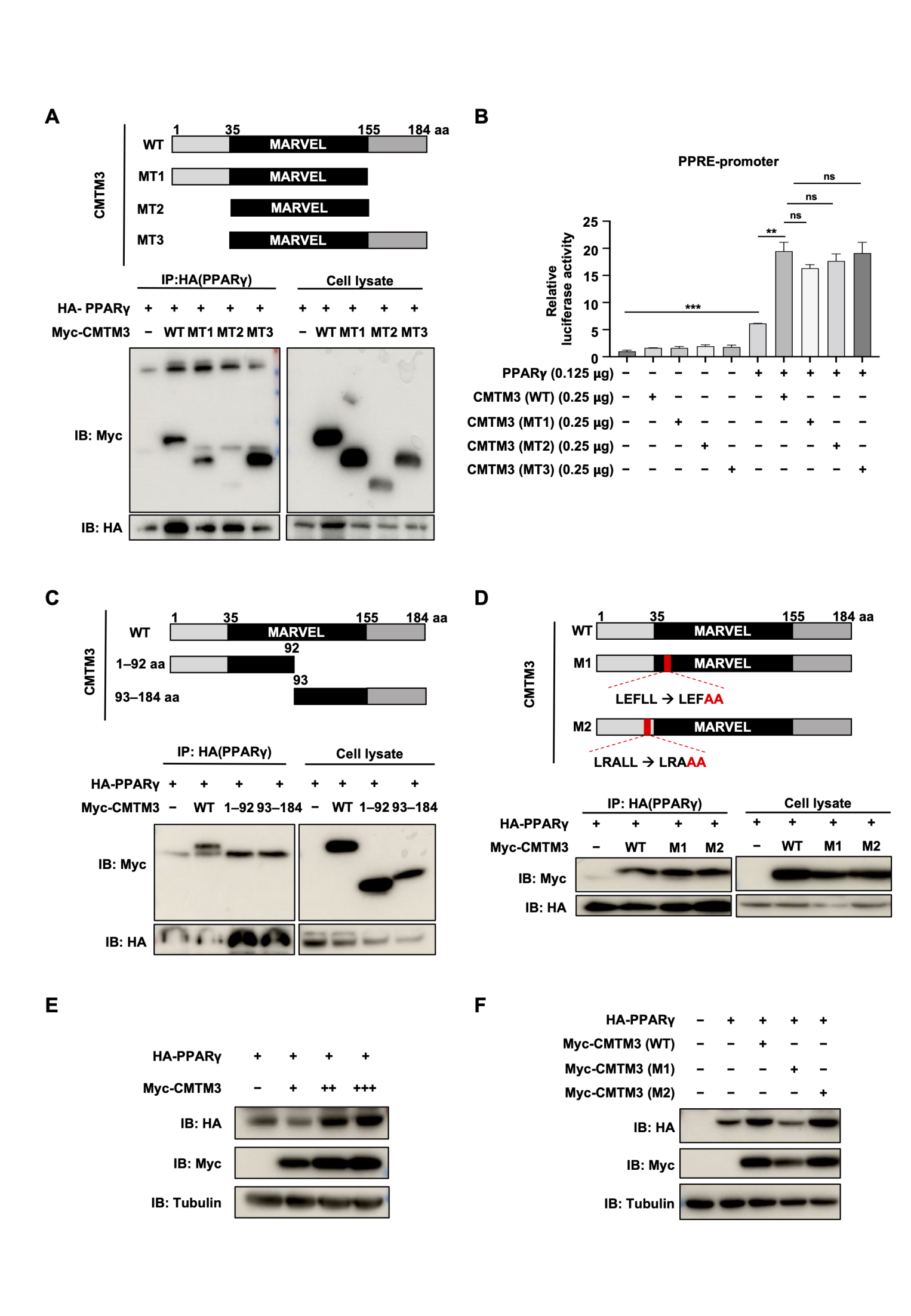
**

**Supplementary Figure 4.** The MARVEL domain of CMTM3 has an important role in CMTM3 function. **(A)** The schematic diagram of CMTM3 deletion. MT1 (ΔC-terminal domain), MT2 (ΔNC), MT3 (ΔN-terminal domain). HEK 293 cells were transfected with an indicated combination of PPARγ, WT of CMTM3, and deleted-CMTM3 form. Co-IP was performed with anti-HA. IB was detected using anti-Myc and anti-HA antibodies. **(B)** The PPRE promoter activity was performed using the luciferase assay. HEK 293 cells were transfected with an indicated combination of PPARγ (0.125 µg**)**, WT of CMTM3 (0.25 µg**)**, and deleted-CMTM3 form (0.25 µg**)**. Data are expressed as mean ± SEM of at least three experiments. ns, not significant. ** *p* < 0.01, *** *p* < 0.001. **(C)** The schematic diagram of CMTM3 deletion (1–92 aa, 93–184 aa). Co-IP was used to detect the interaction between PPARγ and CMTM3 deletion. Co-IP was performed using anti-HA, and IB was conducted using anti-Myc and anti-HA. **(D)** The schematic diagram of the CMTM3 point mutation. M1 (mutation of LEFLL, located in the MARVEL domain); M2 (mutation of LRALL, located in the N-terminal domain). HEK 293 cells were transfected with an indicated combination of PPARγ and CMTM3 (WT, M1, and M2). Co-IP examined the interaction between PPARγ and CMTM3 (WT, M1, and M2) using anti-HA, and IB was conducted using anti-Myc and anti-HA. **(E)** HEK 293 cells were transfected with HA-PPARγ (0.125 µg**)** and increasing amounts of CMTM3 (0.125, 0.25, 0.5 µg**)**. The protein levels were evaluated by IB using anti-HA and anti-Myc. Tubulin was used as the loading control. **(F)** HEK 293 cells were transfected with an indicated combination of HA-PPARγ and Myc-CMTM3 (WT, M1, M2). The protein levels were evaluated by IB using anti-HA and anti-Myc. Tubulin was used as the loading control.
